# The Challenge of Disease-Modifying Therapies in Parkinson’s Disease: Role of CSF Biomarkers

**DOI:** 10.3390/biom10020335

**Published:** 2020-02-19

**Authors:** Federico Paolini Paoletti, Lorenzo Gaetani, Lucilla Parnetti

**Affiliations:** Section of Neurology, Department of Medicine, University of Perugia, 06132 Perugia, Italy; federico.paolinipaoletti@gmail.com (F.P.P.); lorenzo.gaetani@unipg.it (L.G.)

**Keywords:** Parkinson’s disease, neuronal death mechanisms, targets for neuroprotection, genetic risk factor

## Abstract

The development of disease modifying strategies in Parkinson’s disease (PD) largely depends on the ability to identify suitable populations after accurate diagnostic work-up. Therefore, patient molecular profiling and disease subtyping are mandatory. Thus far, in clinical trials, PD has been considered to be a “single entity”. Conversely, in front of the common feature of nigro-striatal degeneration, PD is pathogenically heterogeneous with a series of several biological and molecular pathways that differently contribute to clinical development and progression. Currently available diagnostic criteria for PD mainly rely on clinical features and imaging biomarkers, thus missing to identify the contribution of pathophysiological pathways, also failing to catch abnormalities occurring in the early stages of disease. Cerebrospinal fluid (CSF) is a promising source of biomarkers, with the high potential for reflecting early changes occurring in PD brain. In this review, we provide an overview on CSF biomarkers in PD, discussing their association with different molecular pathways involved either in pathophysiology or progression in detail. Their potential application in the field of disease modifying treatments is also discussed.

## 1. Introduction

Parkinson’s disease (PD) is the most common degenerative movement disorder, with a negative impact on daily-life functioning [1]. Currently available therapies are only symptomatic and they aim at controlling signs and symptoms for as long as possible. No disease-modifying strategies are available so far, and therapies that are able to stop the disease progression are urgently needed. Several clinical trials have been carried out in the last decades with the aim of investigating potential disease-modifying properties of symptomatic drugs. Selegiline and l-dopa (see Deprenyl And Tocopherol Antioxidative Therapy of Parkinsonism (DATATOP) and Earlier versus Later Levodopa Therapy in Parkinson Disease (ELLDOPA) trials, respectively) [2,3] failed to demonstrate neuroprotective abilities, mostly due to their confounding symptomatic effects. Even when delayed-start trials were designed with the aim of overcoming such a limit, rasagiline and l-dopa were not found to slow-down PD progression (see Attenuation of Disease Progression with Azilect Given Once-daily (ADAGIO) trial and Levodopa in Early Parkinson’s Disease (LEAP) study, respectively) [4,5]. More recently, the increasing knowledge about genes and molecular pathways that are involved in PD pathogenesis has led to a revolutionary approach in the development of potential disease-modifying drugs [6]. Mutations and multiplications of the *SNCA* gene coding for α-synuclein were discovered as causes of dominantly inherited PD more than 20 years ago. Since then, a series of other genes, including glucocerebrosidase (*GBA*) and leucine-rich repeat kinase (*LRKK2*) genes, were identified as being responsible for inherited forms of PD [7]. Furthermore, with the development of genome-wide association studies (GWAS) and other novel technologies, it became clear that genetic factors are not only involved in classically Mendelian inherited PD, but also increase the susceptibility to the most common form of sporadic PD. These findings enabled therapies targeting specific molecular pathways to be tested in animal models and consequently in clinical trials. However, biomarkers objectively evaluable as indicators of biological and molecular processes are needed, in order to understand whether a specific pharmacological intervention might be suitable for certain patients to slow-down disease progression. In this sense, cerebrospinal fluid (CSF) and its biomarkers can provide a realistic picture of pathological processes that occur in the brain. This review summarizes recent advances in disease-modifying strategies targeting molecular pathways that are involved in PD pathogenesis, focusing on their current limits and on how CSF biomarkers can be helpful in overcoming such limits. 

## 2. Molecular Targets for Disease-Modifying Strategies in PD

### 2.1. α-Synuclein

**Figure 1:**
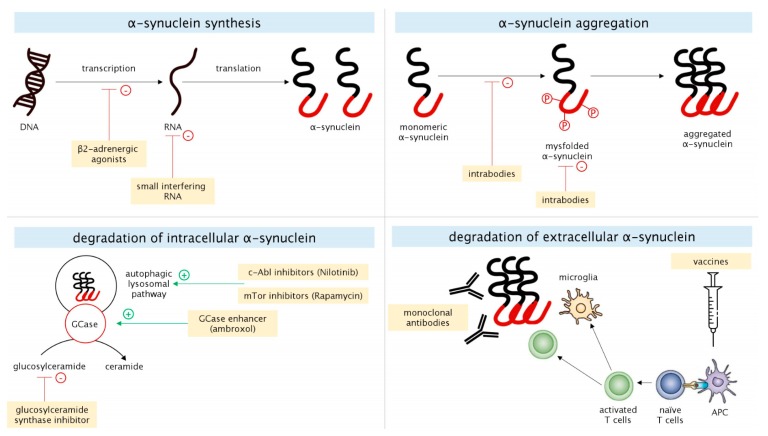
Schematic representation of molecular mechanisms related to α-synuclein metabolism (synthesis, aggregation, intracellular degradation, and extracellular degradation) and potential disease-modifying strategies targeting these mechanisms.

α-Synuclein is a 140-aminoacid protein that is highly expressed in the central nervous system (CNS) and it is mainly located at a presynaptic level. Its functions are not fully understood, but it seems to be involved in vesicles trafficking, neurotransmitters release, membranes remodeling, and synaptic plasticity. A series of genetic and acquired factors can promote misfolding and aggregation of α-synuclein with the subsequent formation of oligomers, amyloid-like fibrils, and Lewy bodies (LBs). Misfolded species of α-synuclein are crucially involved in the pathogenesis of PD, and LBs are considered to be a marker of neuronal degeneration, since the sites of their accumulation correspond to those of neuronal loss in PD. However, increasing evidence suggests that oligomeric (o-α-syn) and fibrillary α-synuclein are also cytotoxic, mainly due to their ability to increase membrane permeability [8]. α-synuclein pathology might be therapeutically targeted in different pathophysiological mechanisms, such as in its expression, aggregation, and degradation (Figure 1). 

#### 2.1.1. α-Synuclein Synthesis 

Oligonucleotide molecules targeting α-synuclein via the RNA interference have been tested both in neuron-like cell cultures and in animal models. In monkeys, unilateral infusion of small interfering RNA (siRNA) reduced the α-synuclein levels of 40–50% when compared to the untreated side [9]. In rodent models, viral-vector derived siRNA were able to suppress 35% of the expression of endogenous α-synuclein [10]. Antisense oligonucleotides reduced α-synuclein production in rats, by promoting ribonuclease H-mediated degradation of α-synuclein messenger RNA [11]. Reducing the transcription of *SNCA* gene coding for α-synuclein represents another possible strategy. β2-adrenergic agonists, such as salbutamol and clenbuterol, can suppress α-synuclein transcription by modulating histone acetylation at the promoter and enhancer regions of the *SNCA* gene [12]. 

#### 2.1.2. α-Synuclein Aggregation 

Intrabodies are small antibodies that are able to enter the cell, bind to monomeric α-synuclein, and prevent its oligomerization. They were found to reduce α-synuclein aggregation and nigro-striatal degeneration in rodent models with viral vector-mediated α-synuclein overexpression [13]. In this field, NPT200-11 and NPT088 are two candidates in current clinical testing phase. A single ascending dose study with orally administered NPT200-11 capsules (from 15 to 480 mg) in healthy subjects was carried out in 2016 to determine the safety, tolerability, blood levels, and maximally tolerated dose of the drug (CinicalTrial.gov identifier NCT02066682). NPT088 is a fusion protein between human immunoglobulin and GAIM (General Amyloid Interaction Motif) protein [14], which was reported to reduce α-synuclein aggregation and protect nigro-striatal neurons (ClinicalTrial.gov identifier NCT03008161). Heat shock proteins (HSP) represent an alternative strategy against α-synuclein aggregation. They are able to stabilize partially folded protein intermediates and maintain cellular proteostatis under stress conditions [15]. Recently, Taguchi et al. demonstrated that the overexpression of HSP110 is sufficient for reducing α-synuclein aggregation in mammalian cell culture models, and it effectively mitigates α-synuclein pathology in mouse models [16].

#### 2.1.3. Degradation of Intracellular α-Synuclein 

The autophagic-lysosomal pathway (ALP) represents one of the main mechanisms by which oligomeric and pro-aggregating species of α-synuclein can be degraded. c-Abl (Abelson tyrosine kinase) is a member of the Abl family of non-tyrosine kinase receptors. C-Abl inhibitors, which have been already approved as treatments for different forms of leukemia, are under investigation as disease-modifying strategies for synucleinopathies. The preclinical findings suggested that c-Abl inhibitors are able to enhance ALP, thus promoting degradation of intracellular α-synuclein, which gave a strong impulse for testing these molecules in clinical trials [17]. Pagan et al. demonstrated that the c-Abl inhibitor Nilotinib penetrates the blood-brain barrier and improves the clinical outcomes of patients suffering from PD with dementia (PDD) and dementia with Lewy bodies (DLB) [18]. More recently, the same group found that Nilotinib enters the CNS in a dose-independent manner, with 200 mg appearing to be an optimal single dose that concurrently reduces inflammation and impacts on CSF biomarkers, including dopamine metabolites and α-synuclein [19]. The serine/threonine kinase mTOR (mammalian target of rapamycin) is a key determinant of the activity of ALP, with its activation inhibiting autophagy. Hence, mTOR inhibitors, including rapamycin and MSDC-0160, have been demonstrated to enhance autophagy and reduce α-synuclein toxicity in preclinical systems [20,21]. 

#### 2.1.4. Degradation of Extracellular α-Synuclein

Immunotherapy, including both active (immune system stimulation) and passive (direct antibodies administration) strategies, is under investigation for PD and it seems to be a promising approach to reduce extracellular α-synuclein. PRX002 is a humanized IgG1 monoclonal antibody directed against epitopes near the C-terminus of α-synuclein [22,23], whereas BIIB054 is a fully human IgG1 monoclonal antibody directed at an epitope near the N-terminus of α-synuclein [24,25]. Further anti-α-synuclein monoclonal antibodies, such as MEDI1341 and BAN0805, are in earlier phases of clinical testing. With regard to active immunotherapies, AFFITOPE is the only one appearing in clinical settings. It is a synthetic vaccine that is characterized by an α-synuclein-mimicking epitope to provide an immune response against α-synuclein [26]. 

### 2.2. GBA

The *GBA* gene codes for the lysosomal enzyme β-glucocerebrosidase (GCase), which catalyses the hydrolysis of glucosylceramide (GluCer) into glucose and ceramide [27]. Homozygous or compound heterozygous mutations in the *GBA* gene cause Gaucher’s disease (GD), a lysosomal storage disorder with an autosomal recessive inheritance [28], whereas heterozygous mutations represent the most common genetic risk factor for PD [27]. The exact mechanism by which *GBA*-carriers have a higher risk of developing PD or other synucleinopathies is not fully understood. However, it is ascertained that GCase is indirectly involved in α-synuclein degradation. Reduced GCase activity leads to higher levels of GluCer, which, in turn, stabilizes the aggregates of α-synuclein and promotes cell-to-cell spreading of misfolded α-synuclein [29]. Aggregates of misfolded α-synuclein hamper vesicular trafficking into the cells, further reducing GCase activity and enhancing α-synuclein accumulation [29]. The existence of a pathophysiological vicious cycle between GCase dysfunction and α-synuclein aggregation is in line with the finding of lower GCase activity in the brain homogenates of PD patients, independently of their *GBA* carrier status. Thus, potential disease-modifying strategies enhancing *GBA*-mediated pathway of α-synuclein degradation are now under investigation [30].

#### 2.2.1. GCase Activity 

The potentiation of GCase activity has been investigated by either gene therapy or small molecular chaperones entering the blood-brain barrier. In the first case, preclinical studies showed that *GBA* augmentation via adeno-associated virus reduced α-synuclein aggregation and cognitive deficits in the mice models of GD-related synucleinopathy and decreased α-synuclein levels in *SNCA* mice models [31]. This evidence suggests that increasing GCase activity can modulate α-synuclein processing and slow-down progression of both *GBA*-related and non *GBA*-associated synucleinopathies. In the second case, two phase-2 clinical trials (ClinicalTrials.gov Identifiers NCT02941822 and NCT02914366) have been started in patients with PD and PDD to investigate ambroxol, which is a mucolytic molecule with chaperon properties that was demonstrated to enhance GCase activity and reduce α-synuclein phosphorylation at Serine-129 in mice and non-human primates [32]. Other small molecules entering the blood-brain barrier are represented by inhibitory chaperones that stabilize GCase by interacting with the enzyme active site (isofagomine) [33] and non-inhibitory chaperones that stabilize GCase by binding to it in other sites and inducing conformational changes (NCGC758 and NCGC607) [34,35]. In induced pluripotent stem cells (iPSC)-derived dopaminergic neurons from PD patients and GD patients, treatment with NCGC758 and NCGC607 increased lysosomal activity and reduced α-synuclein levels, also reverting the subcellular pathologies downstream to α-synuclein accumulation. Recently, another small molecule, called S-181, has been tested in iPSC-derived dopaminergic neurons from sporadic PD patients and from *GBA*-carrier PD patients. In these models, S-181 partially restored lysosomal function and lowered the accumulation of oxidized dopamine, GluCer, and α-synuclein. Similar findings were also obtained treating mice carrying *GBA* heterozygous mutations [36].

#### 2.2.2. *GBA*-Related Glycosphingolipids Metabolism 

The inhibition of synthesis of glycosphingolipids promoting the aggregation and spreading of misfolded α-synuclein species represents an alternative strategy to act on *GBA*-related pathway. GZ667161 (Venglustat, Framingham, MA, USA), a brain-penetrant molecule, inhibits glucosylceramide synthase and reduces the levels of GluCer. This compound was tested in GD-related synucleinopathy and *SNCA* mice models. It was found to slow-down the accumulation of hippocampal aggregates of α-synuclein, ubiquitin and tau, also ameliorating cognitive dysfunctions [37]. A phase-2 clinical trial (ClinicalTrials.gov identifier NCT02906020) in early stage PD patients carrying *GBA* mutations is currently ongoing, for evaluating the efficacy of GZ667161 in reducing motor and cognitive decline. 

### 2.3. LRRK2

The mutations in the *LRRK2* gene represent the most frequent genetic cause of dominantly inherited PD [7]. Five different domains—including a kinase domain—each of them with specific functions, characterize LRRK2 protein. It is involved in a series of different molecular pathways including ALP, maintenance of actin skeleton, vesicle trafficking, and immune responses [38]. LRRK2 is specifically involved in the ALP-related degradation of α-synuclein. G2019S mutation in *LRRK2* increases its kinase activity and enhances the efficiency of misfolded α-synuclein spreading [39]. Furthermore, LRRK2 is implicated in the intrinsic regulation of microglial activation, which is believed to contribute to neuroinflammation and neuronal death in PD [40]. Thus, LRRK2 interacts with many key proteins that are implicated in PD, suggesting its pivotal role, even in the pathogenesis of sporadic PD, and offering a new potential target for disease-modifying approaches. Indeed, two phase-1 placebo-controlled clinical trials are ongoing to determine safety, tolerability, pharmacokinetics, and pharmacodynamics of two LRRK2 kinase activity inhibitors, named DNL201 (ClinicalTrials.gov identifier NCT03710707) and DNL151 (ClinicalTrials.gov identifier NCT04056689). 

### 2.4. Other Molecular Targets

There is considerable interest in potential disease-modifying strategies targeting molecular mechanisms other than those that are related to α-synuclein, Gcase, and LRRK2 pathways. For instance, a clinical trial testing safety, tolerability, pharmacokinetics, and pharmacodynamics of KM-819, a Fas-associated factor 1 (FAF1) inhibitor, has been recently completed in healthy volunteers (ClinicalTrials.gov Identifier NCT03022799) [41]. FAF1 is related to Fas-mediated apoptosis and its levels are found to be increased in the midbrain of PD animal models. Thus, KM-819 has the potentiality to slow-down PD progression by inhibiting the Fas-mediated cell death pathway [42]. Physiopathology underlying PD development is also linked to exaggerating oxidative stress, mitochondrial dysfunction, and aberrant inflammatory activation. EPI-589 (also known as [R]-troloxamide quinone) is an oxidoreductase enzyme inhibitor, being able to increase the subcellular levels of the antioxidant glutathione (GSH). GSH deficiency in the brain is known to be implicated in mitochondrial dysfunctions leading to PD. EPI-589 has been already found to be safe and well tolerated, with effects on biomarkers of neuroinflammation and on disease progression in patients with amyotrophic lateral sclerosis [43]. A phase-2 clinical trial has been recently carried out to determine whether EPI-589 can alter the biochemical signature of PD, as assessed by peripheral blood and brain imaging biomarkers (ClinicalTrials.gov Identifier NCT02462603). In the field of inflammation-related oxidative stress, AZD3241 seems to be one of the most promising molecules as potential disease-modifying drug. It is a selective and irreversible inhibitor of myeloperoxidase, an enzyme that is highly expressed by microglia and able to generate large amounts of reactive oxygen species. In a recent phase-2 randomized placebo controlled trial, AZD3241 was found to reduce neuroinflammation in a small group of PD patients after four weeks of treatment, as assessed by positron emission tomography (PET) imaging (ClinicalTrials.gov Identifier NCT01527695) [44]. These are only few examples of alternative targeting mechanisms for disease-modifying strategies. Several studies investigating the treatments against oxidation and neuroinflammation either have been completed or are ongoing, with still unreported results [45]. Overall, each of the previously discussed targeting mechanisms should not be considered singularly and segregated from each other. Abnormalities occurring in one of these pathways are able to trigger dysfunctions in others pathways, rising up a self-sustained vicious cycle.

## 3. CSF Biomarkers: An Overview

CSF is strictly in contact with neurons and, therefore, it is a source of biomarkers potentially offering the most promising insight into the pathogenesis of neurodegenerative disorders. CSF biomarkers have been extensively investigated in terms of both diagnostic and prognostic value in PD. 

While considering its fundamental role in PD pathogenesis, great attention has been paid to α-synuclein. The majority of studies showed lower CSF total α-synuclein levels in PD patients when compared to control subjects [46,47,48], probably reflecting its pathological sequestration into LBs. However, other independent studies either found no differences in CSF α-synuclein between PD patients and healthy subjects [49], or indicated that CSF α-synuclein tends to increase along the disease course [50,51]. The divergence of these results are linked, in part, to the fact that α-synuclein is physiologically located at a presynaptic level and its concentrations in the CSF can reflect not only its deposition in LBs, but also its release from degenerating synapses. Misfolded species of α-synuclein including oligomeric and phosphorylated α-synuclein seem to be increased in the CSF of PD patients, but their application as biomarkers is hampered by some limitations mainly dealing with their lower concentrations as compared to monomeric α-synuclein and the lack of selective antibodies with high affinity and avidity to these species [52,53]. 

Post-mortem studies showing the coexistence of amyloid and tau pathologies with LBs in synucleinopathies have led to investigating core Alzheimer’s disease (AD) biomarkers—amyloid beta 40 (Aβ40) and amyloid beta 42 (Aβ42) proteins, total tau, and phosphorylated tau proteins—in PD [54]. Several studies highlighted the prognostic role of Aβ42 in terms of cognition since lower CSF Aβ42 levels in PD patients at baseline predict earlier appearance of cognitive decline [55]. Conversely, more doubtful results were found when CSF tau proteins were studied as possible predictive factors for cognitive impairment. Most of the longitudinal studies failed to show any association between tau proteins and cognition in PD. Being increased in AD, stroke and encephalitic diseases, total tau protein is a quite unspecific marker of neuronal injury reflecting the extent of degenerative processes, rather than tau pathology itself. It seems to be lower in PD patients compared to healthy subjects, and increased in atypical parkinsonisms that are characterized by a more rapid and aggressive neurodegeneration [56]. Of interest, recent evidence suggests that in the CSF, tau is also present as different fragments that, contrarily to total tau protein, could be disease-specific for primary tauopathies, such as progressive sopranuclear palsy (PSP) and corticobasal syndrome (CBS) [57]. 

Neurofilament light chain (NfL) represents another marker of large myelinated axonal damage. Similarly to total tau protein, it does not increase in the CSF of PD patients, probably due to a relative sparing of largest myelinated axons at least in the earliest stages of the disease [58]. Otherwise, it sharply increases in PSP, CBS, and multisystem atrophy, thus showing good accuracy in discriminating PD from atypical parkinsonisms [59]. 

In line with the pathophysiological role of GCase, which, when decreased, leads to α-synuclein pathology, GCase activity has been repetitively found to be decreased in the CSF of PD patients. However, its diagnostic accuracy is poor when considered alone, and it was improved by combining either other lysosomal enzymes or α-synuclein and Aβ42 [47,60].

A series of other CSF biomarkers are under investigation in PD. They include markers that are related to inherited forms of PD (LRRK2 and DJ-1) [61] and inflammatory markers (e.g., YKL-40, MCP-1 and cytokines) [62]. 

Further potential CSF biomarkers for PD have been recently evidenced in a few studies that are based on proteomics and metabolomics. These approaches represent an emerging field still suffering from several limitations that are linked to both pre-analytical and analytical factors [63].

## 4. Disease-Modifying Therapies in PD: Challenges, Open Issues and Potential Role of CSF Biomarkers

**Figure 2:**
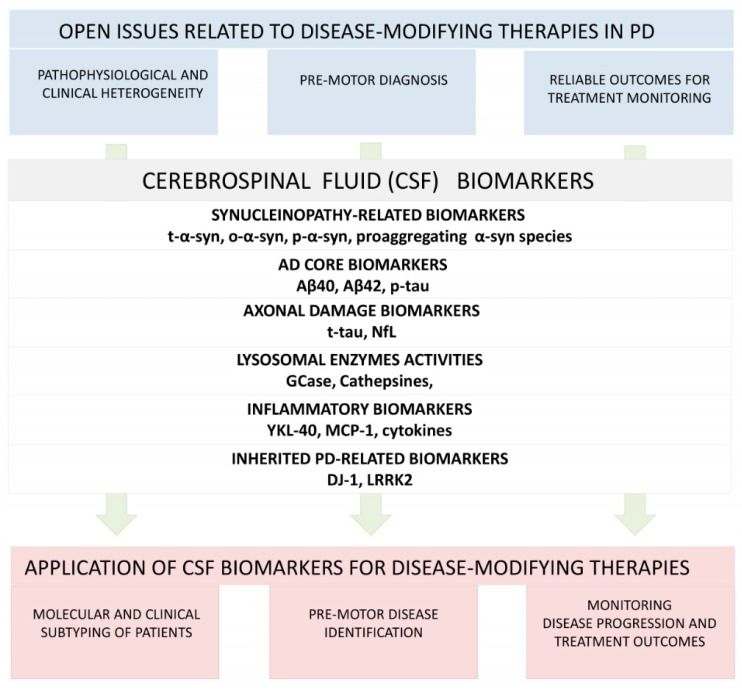
The figure shows a series of cerebrospinal fluid biomarkers which can be potentially used to face up current limits and open issues related to disease-modifying strategies for Parkinson’s disease. Aβ-40: amyloid beta 40 protein; Aβ-42: amyloid beta 42 protein; α-syn: α-synuclein; GCase: β-glucocerebrosidase; LRRK2: leucine-rich repeat kinase 2; MCP-1: monocyte chemoattractant protein 1; NfL: neurofilament light chain; o-α-syn: oligomeric α-synuclein; p-α-syn: phosphorylated α-synuclein; PD: Parkinson’s disease; p-tau: phosphorylated tau protein; t-α-syn: total α-synuclein; t-tau: total tau protein; YKL-40: Chitinase-3-like protein 1.

Some of the clinical trials evaluating drugs for potential disease-modifying properties in PD have not reached satisfactory results. The reasons underlying these results might be different, including the pathophysiological and clinical heterogeneity of PD and the difficulty in detecting pre-motor PD, as well as the lack of objective and reliable outcomes to evaluate drug’s efficacy. Different CSF biomarkers show the potentiality of overcoming such limits (Figure 2). 

### 4.1. The Issue of Pathophysiological and Clinical Heterogeneity

Over the last few years, the complexity of PD has led to the concept of a spectrum of different disorders rather than a single disease [64]. From a clinical point of view, different phenotypic subtypes exist, with both different symptoms at the onset and different progression rates. Additionally, from a pathophysiologic point of view, in front of the common feature on nigro-striatal degeneration, PD is heterogeneous, with a series of several biological and molecular pathways differently contributing to its development and progression. Besides the aggregates of misfolded α-synuclein and Lewy bodies, mitochondrial dysfunction, oxidative stress, ALP disorders and inflammatory responses can be involved, and each of them is variably expressed among different PD subtypes [65]. Even the presence of α-synuclein aggregates and Lewy bodies has been questioned as constant feature and cause of nigro-strial degeneration. Indeed, patients with *LRRK2* mutations, instead of classical Lewy body pathology, may exhibit either nigral degeneration with tau inclusions resembling progressive sopranuclear palsy or α-synuclein pathology in the form of glial cytoplasmic inclusions, like in multisystem atrophy [66]. Thus, a specific drug can act on most of patients only if the targeted biological mechanism is sufficiently represented in the selected population. For instance, symptomatic dopaminergic replacement therapy is useful, since dopamine deficiency mainly due to nigro-striatal degeneration is common in all PD patients. However, should a mitochondrial enhancer drug be tested, it would be fundamental to select a population of PD patients, in which mitochondrial dysfunctions are predominant and represent a pivotal event in the pathogenesis of their disease. Furthermore, preclinical—both animal and cellular—models are not able to reflect the complex pathogenesis of PD, which can explain how the auspicious and successful findings from preclinical research have not been translated in equivalent progresses in terms of clinical practice [67]. Most of preclinical models reflect a single predominant pathogenic mechanism, without considering the interplay between different molecular pathways that occur in human brain; they develop changes quite acutely and do not represent the progression of pathology from prodromal to clinical stages. 

#### Role of CSF Biomarkers 

Defining PD subtypes is essential to select and enroll specific groups of patients in clinical trials for disease-modifying therapies. The clinical and imaging features are often insufficient. Further information coming from CSF biomarkers can be helpful in defining the complex heterogeneity of PD. One of the most recent and successful attempts to characterize a huge population of PD patients is derived from the Parkinson’s Progression Markers Initiative (PPMI), an ongoing international multicenter prospective study to validate biomarkers in early and drug-naïve PD patients. When CSF biomarkers (α-synuclein, Aβ42, and total and phosphorylated tau proteins) were correlated with clinical features of more than 600 PPMI subjects at baseline, lower α-synuclein levels were found in patients with non-tremor dominant phenotype as compared to those with tremor-dominant phenotype [68]. A similar result was also obtained in moderate-advanced PD patients from the BioFIND study that revealed lower CSF α-synuclein levels in patients with postural instability-gait disturbances phenotype when compared to other motor phenotypes [69]. Furthermore, in the PPMI population, lower Aβ42 levels and higher total tau/Aβ42 were found in patients with more severe non-motor symptoms [68]. Particularly, Aβ42 showed a significant correlation with cognition in PD in different studies, also being regarded as an independent risk factor, at baseline, for the development of cognitive decline in PD patients [55]. In a cohort of PD patients (both non-demented and demented) and controls, CSF Aβ42 ranged from high (controls) to intermediate (non-demented) and low (demented) levels [70,71]. More recently, 223 PD patients from the PPMI were classified into three different subtypes while using a cluster analysis approach, in which a motor summary score and three non-motor features—cognitive impairment, REM behavioral disorders (RBD), and dysautonomia—were considered: (1) mild-motor predominant, (2) intermediate, and (3) diffuse malignant subtypes. Patients with diffuse malignant PD showed the lowest levels of Aβ42 and Aβ42/total tau ratio and they progressed faster in overall prognosis with greater decline in cognition and in dopamine functional imaging after an average of almost three years [72]. Overall, these findings suggest that α-synuclein pathology can mainly influence motor phenotype, whereas cognition and other non-motor characteristics are mostly driven by amyloid and, to a less extent, tau pathologies. Thus, concerning disease-modifying strategies, a hypothetical experimental drug that is designed to influence non-tremor dominant phenotype should be tested on patients with the lowest CSF α-synuclein concentration. Similarly, experimental drugs for cognitive impairment should be tested on patients with lower CSF Aβ42 levels.

### 4.2. The Issue of Pre-Motor Diagnosis 

The cardinal motor features of PD appear when the majority of dopaminergic nigro-striatal neurons are lost. This has led to the concepts of preclinical and prodromal PD. Preclinical PD refers to the condition in which molecular aberrations and pathological changes have already started, but subjects are asymptomatic, while prodromal PD indicates the phase in which non-motor symptoms or subtle motor signs are present without fulfilling the diagnostic criteria for clinically established PD [73]. The pre-motor phase of neurodegeneration is of utmost importance for the development of disease-modifying strategies, which should be applied as earlier as possible. The disease could be pathogenically too advanced, even in the early symptomatic patients, thus making it difficult to obtain a benefit from treatments designed against putative pathogenic mechanisms. Furthermore, it seems quite difficult to understand in which phase of disease each biological mechanism of pathogenesis happens, making it even more challenging to predict the effects of disease-modifying therapies. 

#### Role of CSF Biomarkers 

CSF biomarkers that are able to identify PD in its pre-motor stages are urgently needed with a view of developing disease-modifying strategies. Data regarding CSF biomarkers for preclinical and prodromal PD are very scarce, since asymptomatic subjects at risk of developing PD (for instance subjects with genetic mutations for familial PD) and patients with prodromal symptoms (for instance patients with hyposmia or RBD) are rarely recruited in studies assessing CSF. Compta et al. [74] measured CSF α-synuclein levels in healthy controls, RBD, non-demented PD, and PDD patients, with the aim of assessing the prodromal-motor-dementia continuum of PD. Whereas both total α-synuclein and oligomeric/total ratio did not significantly differ among groups, oligomeric α-syn showed higher levels in both PD and PDD patients when compared to RBD patients and controls. Seeding aggregation assays including Real-Time Quaking Induced Conversion (RT-QuIC) and Protein-Misfolding Cyclic Amplification (PMCA) are unconventional techniques recently investigated to detect species of α-synuclein prone to aggregation in the CSF of patients affected by synucleinopathies. Shahnawaz et al. [75] analyzed, by means of PMCA, CSF samples from PD patients, AD patients, and individual serving as controls that are affected by other neurologic disorders. They correctly identified patients who were affected by PD with overall sensitivity of 88.5% and specificity of 96.9%. It is worth noting that two samples belonging to controls at baseline, resulted positive; some years later, these patients developed PD. Similarly, by using RT-QuIC, Fairfoul et al. identified α-synuclein aggregation in CSF samples from PD and DLB patients with sensitivities of 92% and 95%, respectively, and with an overall specificity of 100% as compared to AD patients and controls [76]. They obtained positive results, even when they tested CSF samples from RBD patients, thus suggesting the possibility of identifying synucleinopathies at prodromal stages. Nowadays, a great effort is done in order to implement these very promising approaches. 

### 4.3. The Issue of Reliable Outcomes for Treatment Monitoring

The lack of reliable biomarkers for assessing both disease-related severity and impact of therapy is one of the most challenging issues in the field of disease-modifying strategies. The currently available outcome measures mainly refer to clinical and imaging markers that suffer two main biases. Firstly, they can be affected by a series of factors unrelated to degenerative process itself and including the symptomatic effects of experimental drugs and the misinterpreted disease-modifying effects of conventional dopaminergic medications. Secondly, these markers largely reflect the nigro-striatal pathway that might be poorly influenced by the experimental molecules tested in clinical trials. Other factors that need to be critically considered in clinical trials are represented by the impact of drugs on compensatory processes and concurrent pathologies, as well as the potential molecular effects on specific imaging markers, such as ^123^I-Ioflupane Single Photon Emission Computed Tomography (I-FP-CIT SPECT) (e.g., pharmacodynamics could influence the affinity of the ligand for its target) [45].

#### Role of CSF Biomarkers 

The identification of biomarkers reflecting disease progression is mandatory for obtaining outcome measures for clinical trials and testifying the impact of experimental drugs on disease. CSF provides a better representation of pathological aberrations occurring in the CNS, as compared to other peripheral fluids, also monitoring their changes along time. Thus, the interest in CSF biomarkers in PD has progressively involved not only the diagnostic assessment, but also its characterization in terms of progression. A few works reported lower CSF total α-synuclein [77,78] and higher oligomeric α-synuclein levels in the more advanced stages of PD, as assessed by Hoehn and Yahr stages [79]. More precious information can be obtained by longitudinal studies. Majbour et al. assessed longitudinal CSF levels of different species of α-synuclein in a cohort of 121 patients from the DATATOP study and found that the total and oligomeric α-synuclein levels significantly increased along the two-year follow-up, whereas phosphorylated α-synuclein showed a longitudinal decrease [50]. Moreover, they noted an association between the change of oligomeric/total α-synuclein ratio and the worsening of motor signs, in particular in the postural instability and gait disturbances (PIGD)-dominant PD group. Conversely, in another independent cohort from the DATATOP study, CSF levels of phosphorylated α-synuclein increased over two years of disease progression and negatively correlated with Unified PD Rating Scale (UPDRS) motor scores only at the baseline, suggesting that this association depends on disease stage [80]. In two independent cohorts from the Swedish BioFinder study, higher baseline levels of total α-synuclein were associated with greater progression of motor symptoms over two years [81] and a longitudinal increase of total α-synuclein was found after two-year follow-up in PD patients with longer disease duration (>5 years) [51]. Divergent results were obtained when the activities of lysosomal enzymes were tested in the CSF of PD patients. GCase activity positively correlated with both severity of motor impairment and disease stage in PD, with lower values in the early stages, in a study assessing CSF of 71 PD patients [47]. Otherwise, a significant decrease in GCase and cathepsin D activities was observed in the more advanced stages of PD in an independent cohort of 79 patients from the BioFIND study, also showing a positive association between GCase activity and cognitive performances tested by Montreal Cognitive Assessment [60]. Further and larger longitudinal studies are hopefully needed to clarify this divergence of results before CSF biomarkers could be largely used as outcome measures in clinical trials.

### 4.4. State of Art of Ongoing Clinical Trials Adopting CSF Biomarkers as Outcome Measures

A series of CSF biomarkers have been used as outcome measures in different PD clinical trials (Table 1); some of them are now completed with available results. Particularly, α-synuclein, perhaps due to its involvement in one of the main molecular pathways linked to PD pathogenesis, is the most frequent biomarker that is used as outcome measure. CSF α-synuclein was assessed in the first clinical trial testing efficacy, safety, and tolerability of Nilotinib in PDD patients. Twelve patients were randomized into 150 and 300 mg Nilotinib dosage groups, receiving an oral daily dose for 24 weeks with subsequent 12 weeks follow-up [18]. CSF was collected at baseline, at two months and six months of treatment. CSF α-synuclein was reduced at two and six months when compared to baseline in the 150 mg group, whereas it was unchanged in the 300 mg group between baseline, two, and six months. Similar results were obtained in another clinical trial evaluating pharmacokinetics and pharmacodynamics of Nilotinib [19]. Seventy-five PD patients were randomized into five groups receiving a single daily dose of placebo, 150, 200, 300, and 400 mg of Nilotinib. CSF was obtained from 1 to 4 h after drug administration. No changes were found in the CSF total α-synuclein, whereas oligomeric α-synuclein was significantly reduced in the 400 mg group after 3 h of Nilotinib administration. In a more recent trial testing KM-819, CSF samples were collected on days 1 (at baseline) and 7 (after the last drug administration) [41]. No clear treatment- or dose-related changes from day 1 to day 7 were found in the CSF oligomeric α-synuclein. The dynamic pattern of CSF α-synuclein along treatment with potential disease-modifying drugs, particularly with Nilotinib, needs to be completely elucidated. The decrease of α-synuclein in the CSF strongly depends on its sequestration into LBs. Thus, an optimal drug should increase or stabilize CSF α-synuclein in the CSF, mirroring the attenuation of its accumulation in the brain. However, it should also be considered that α-synuclein, due to its prevailing localization at a presynaptic level, reflects synaptic degeneration and its decreasing levels in the CSF could indicate a reduction of neurodegeneration. The attenuation of degenerative processes is also corroborated by further evidence derived from Nilotinib clinical trials. One is represented by the decrease of total tau protein along treatment. In the clinical trial testing Nilotinib in PDD patients, total tau was significantly lowered at two months in the 150 mg group and at six months in the 300 mg group [18]. The other is related to the markers of neuronal and glial death, including neuron specific enolase (NSE) and S100B, respectively, which were found to progressively decrease after treatment [18]. Furthermore, when CSF levels of dopamine (DA) metabolites were used as outcome measures, both homovanillic acid (HVA) and 3,4-dihydroxyphenylacetic acid (DOPAC) were demonstrated to increase along treatment with Nilotinib [18,19], suggesting a possible role for Nilotinib in increasing dopamine levels. It is in agreement with the preclinical findings that Nilotinib protects dopaminergic neurons and increases both DA and HVA in MPTP (1-methyl-4-phenyl-1,2,3,6-tetrahydropyridine) and α-synucleinopathy models of PD [17]. Overall, these findings, in association with the awareness of the complex pathophysiology underlying PD, suggest that a single biomarker, such as α-synuclein, is not sufficient for ascertaining whether a potential disease-modifying drug is able to effectively slow-down neurodegeneration [82]. A panel of different CSF biomarkers should be simultaneously used in clinical trials. 

## 5. Conclusions

The application of CSF biomarkers in the field of degenerative diseases has made a deeper comprehension, in vivo, of biological and molecular processes occurring in the brain. Particularly, in PD, α-synuclein seems to be the biomarker with the highest neuropathological rational of use, since it reflects one of the most important pathophysiological mechanisms driving synucleinopathies. However, α-synuclein levels in the CSF reflect not only its aggregation into LBs, but also its loss from degenerating synapses. Thus, it should be always tested in association with other biomarkers reliably reflecting axonal damage. Nevertheless, most of the studies assessing total tau protein and NfL in the CSF provided inconclusive results, finding no significant differences between PD patients and healthy controls. This is just one of different examples that should prompt us to investigate multiple CSF biomarkers simultaneously. The measurement of a composite panel of biomarkers simultaneously mirroring different biological and molecular pathways seems the best approach to be used in clinical settings since PD is characterized by an intricate interplay of different pathophysiological mechanisms. However, this hypothetical panel should include not only biomarkers that are strictly related to PD pathophysiology (e.g., α-synuclein, lysosomal enzymes, LRRK2, DJ1, and inflammatory markers), but also those biomarkers that are more involved in PD progression (e.g., Aβ42, tau proteins and NfL). 

Given these premises, the potential usefulness of CSF biomarkers in the field of experimental drugs for PD relies on (i) molecular and clinical subtyping of patients to be recruited in clinical trials, (ii) identifying pre-motor disease, which would benefit more from potential disease-modifying therapies and (iii) monitoring disease progression and treatment outcomes. Overall, CSF biomarkers seem to be the most promising outcome measures to be used in clinical trials testing disease-modifying strategies. Nevertheless, diagnostic and prognostic CSF biomarkers for PD have not been completely validated so far, and their use is mainly confined to research purposes. A series of both pre-analytical and analytical limitations need to be considered. The first group includes sample collecting and handling procedures. Most of the studies exploring pre-analytical variability focused on core AD biomarkers. Thus, specific guidelines for CSF collection and handling have been established in the field of AD biomarkers. Otherwise, the identification of the best pre-analytical procedures is still an open issue for other PD candidate biomarkers, such as α-synuclein and lysosomal enzymes. The second group deals with the variability of manufacturing procedures and the techniques for assays to measure CSF biomarkers among different laboratories, which negatively impacts on diagnostic accuracy [83]. The heterogeneity of recruited PD patients and healthy subjects, the small number of studies assessing these biomarkers in prodromal patients, as well as the small number of longitudinal studies represent other limitations. It is mandatory to control these factors and overcome such limits before CSF biomarkers can be accurately used for disease-modifying strategies. 

## Figures and Tables

**Table 1:** Cerebrospinal fluid (CSF) biomarkers adopted as outcome measures in clinical trials for potential disease-modifying therapies targeting molecular mechanisms.

CSF Biomarkers	Targeting Mechanism	Drug	CinicalTrial.gov Identifier	Phase	Recruited Subjects	Period
α-syn (t-α-syn; o-α-syn)	c-Abl inhibition	Nilotinib	NCT022814774	I	12 PDD patients	Nov. 2014–May 2015
		Nilotinib	NCT02954978	II	75 PD patients	Jan. 2017–ongoing
	Anti-α-syn antibody	MEDI1341	NCT03272165	I	48 healthy volunteers	Oct. 2017–ongoing
	Anti-α-syn vaccine	AFFITOPE^®^ PD01A	NCT01568099	I	32 PD patients	Feb. 2012–May 2014
			NCT02216188	I	28 PD patients	Aug. 2014–Aug. 2015
	GCase activation	Ambroxol	NCT02914366	II	75 PDD patients	Nov. 2015–ongoing
	FAF1 inhibition	KM-819	NCT03022799	I	88 healthy volunteers	Oct. 2016–Oct. 2017
Tau proteins (t-tau; p-tau)	c-Abl inhibition	Nilotinib	NCT022814774	I	12 PDD patients	Nov. 2014–May 2015
	GCase activation	Ambroxol	NCT02914366	II	75 PDD patients	Nov. 2015–ongoing
	FAF1 inhibition	KM-819	NCT03022799	I	88 healthy volunteers	Oct. 2016–Oct. 2017
Aβ-42	c-Abl inhibition	Nilotinib	NCT022814774	I	12 PDD patients	Nov. 2014–May 2015
	GCase activation	Ambroxol	NCT02914366	II	75 PDD patients	Nov. 2015–ongoing
GCase activity	GCase activation	Ambroxol	NCT02941822	II	20 PD patients (10 *GBA*+, 10 *GBA*-)	Dec. 2016–Apr. 2018
Neuronal and glial death biomarkers (NSE; S100B)	c-Abl inhibition	Nilotinib	NCT022814774	I	12 PDD patients	Nov. 2014–May 2015
Dopamine metabolites (HVA; DOPAC)	c-Abl inhibition	Nilotinib	NCT022814774	I	12 PDD patients	Nov. 2014–May 2015
	c-Abl inhibition	Nilotinib	NCT02954978	II	75 PD patients	Jan. 2017–ongoing

Aβ-42: amyloid beta 42 protein; α-syn: α-synuclein; c-Abl: Abelson tyrosine kinase; DOPAC: 3,4-dihydroxyphenylacetic acid; FAF1: Fas associated factor 1; GCase: β-glucocerebrosidase; HVA: homovanillic acid; NSE: neuron-specific enolase; o-α-syn: oligomeric α-synuclein; PD: Parkinson’s disease; PDD: Parkinson’s disease with dementia; p-tau: phosphorylated tau protein; t-α-syn: total α-synuclein; t-tau: total tau protein.
